# 9-(4-Methoxy­phen­yl)-3,3,6,6-tetra­methyl-3,4,6,7-tetra­hydro-2*H*-xanthene-1,8(5*H*,9*H*)-dione

**DOI:** 10.1107/S160053680800603X

**Published:** 2008-03-07

**Authors:** Mustafa Odabaşoğlu, Muharrem Kaya, Yılmaz Yıldırır, Orhan Büyükgüngör

**Affiliations:** a Department of Chemistry, Faculty of Arts & Science, Ondokuz Mayıs University, TR-55139 Kurupelit Samsun, Turkey; b Department of Chemistry, Faculty of Arts & Science, Dumlupınar University, Kütahya, Turkey; c Department of Chemistry, Faculty of Arts & Science, Gazi University, Ankara, Turkey; d Department of Physics, Faculty of Arts & Science, Ondokuz Mayıs University, TR-55139 Kurupelit Samsun, Turkey

## Abstract

In the mol­ecule of the title compound, C_24_H_28_O_4_, the three six-membered rings of the xanthene system are not planar, having envelope, boat and envelope conformations. In the crystal structure, C—H⋯O hydrogen bonds link the mol­ecules, generating centrosymmetric *R*_2_^2^(12), *R*_4_^4^(28) and *R*_2_^2^(16) ring motifs and forming a three-dimensional network.

## Related literature

For general background, see: Menchen *et al.* (2003 ▶); Banerjee & Mukherjee (1981 ▶); Nogradi (2003 ▶); Kamel & Shoeb (1964 ▶); Hideo (1981 ▶); Poupelin *et al.* (1978 ▶); Lambert *et al.* (1997 ▶). For ring puckering parameters, see: Cremer & Pople (1975 ▶). For ring motif details, see: Bernstein *et al.* (1995 ▶); Etter (1990 ▶). For related literature, see: Horning & Horning (1946 ▶).
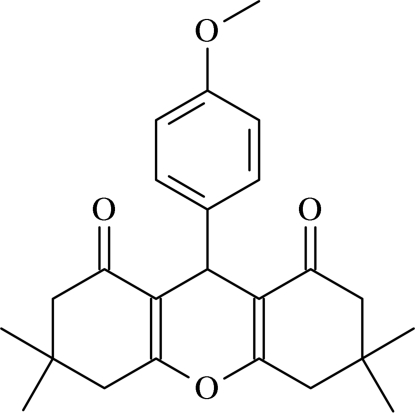


## Experimental

### 

#### Crystal data

C_24_H_28_O_4_
*M* *_r_* = 380.46
Triclinic, 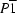

*a* = 9.346 (5) Å
*b* = 10.314 (5) Å
*c* = 11.733 (5) Å
α = 71.089 (5)°
β = 84.253 (5)°
γ = 73.386 (5)°
*V* = 1025.2 (9) Å^3^
*Z* = 2
Mo *K*α radiation
μ = 0.08 mm^−1^
*T* = 296 K
0.66 × 0.54 × 0.41 mm

#### Data collection

Stoe IPDSII diffractometer
Absorption correction: integration (*X-RED32*; Stoe & Cie, 2002 ▶) *T*_min_ = 0.947, *T*_max_ = 0.975
17059 measured reflections
4035 independent reflections
3450 reflections with *I* > 2σ(*I*)
*R*_int_ = 0.033

#### Refinement

*R*[*F* ^2^ > 2σ(*F* ^2^)] = 0.041
*wR*(*F* ^2^) = 0.108
*S* = 1.04
4035 reflections
253 parameters
H-atom parameters constrained
Δρ_max_ = 0.16 e Å^−3^
Δρ_min_ = −0.17 e Å^−3^

### 

Data collection: *X-AREA* (Stoe & Cie, 2002 ▶); cell refinement: *X-AREA*; data reduction: *X-RED32* (Stoe & Cie, 2002 ▶); program(s) used to solve structure: *SHELXS97* (Sheldrick, 2008 ▶); program(s) used to refine structure: *SHELXL97* (Sheldrick, 2008 ▶); molecular graphics: *ORTEP-3 for Windows* (Farrugia, 1997 ▶); software used to prepare material for publication: *WinGX* (Farrugia, 1999 ▶).

## Supplementary Material

Crystal structure: contains datablocks I, global. DOI: 10.1107/S160053680800603X/hk2429sup1.cif

Structure factors: contains datablocks I. DOI: 10.1107/S160053680800603X/hk2429Isup2.hkl

Additional supplementary materials:  crystallographic information; 3D view; checkCIF report

## Figures and Tables

**Table 1:** Hydrogen-bond geometry (Å, °)

*D*—H⋯*A*	*D*—H	H⋯*A*	*D*⋯*A*	*D*—H⋯*A*
C11—H11*A*⋯O4^i^	0.97	2.57	3.364 (2)	139
C15—H15*C*⋯O3^ii^	0.96	2.58	3.506 (2)	161
C22—H22⋯O1^iii^	0.93	2.42	3.343 (2)	171

Symmetry codes: (i) 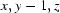; (ii) 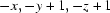; (iii) 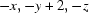.

**Fig. 1.**
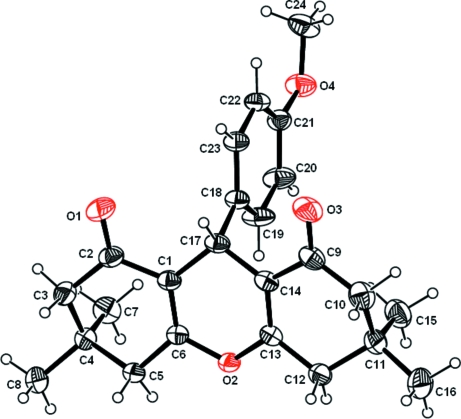
The molecular structure of the title molecule, with the atom-numbering scheme. Displacement ellipsoids are drawn at the 30% probability level.

**Fig. 2.**
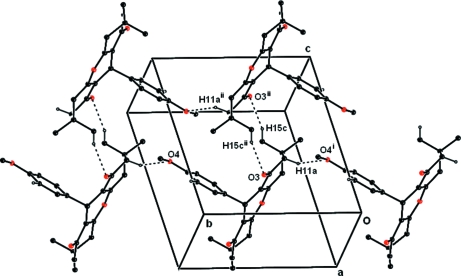
A partial packing diagram of (I), showing the formation of R22(12) and R44(28) ring motifs. Hydrogen bonds are shown as dashed lines [symmetry codes: (i) x, 2 - y, z; (ii) -x, 1 - y, 1 - z]. H atoms not involved in hydrogen bondings have been omitted for clarity.

**Fig. 3.**
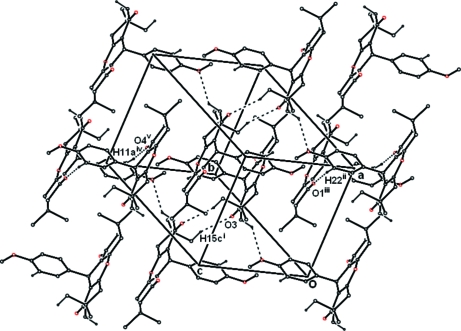
A packing diagram of (I). Hydrogen bonds are shown as dashed lines [symmetry codes: (i) -x, 1 - y, 1 - z; (ii) x + 1, y, z; (iii) x + 1, y + 1, z; (iv) -x, 2 - y, 1 - z; (v) x, y, z + 1]. H atoms not involved in hydrogen bondings have been omitted for clarity.

## supplementary crystallographic information

### Comment

Xanthenes are an important class of organic compounds that find use as dyes, fluorescent materials for visualization of biomolecules and in laser technologies, due to their useful spectroscopic properties (Menchen *et al.*, 2003; Banerjee & Mukherjee, 1981). Oxidation of these compounds can be converted to the corresponding xanthylium salts, which are also effective as dyes and fluorescent materials (Nogradi, 2003; Kamel & Shoeb, 1964). Xanthenes have also received considerable attention from many pharmaceuticals and organic chemists, actually because of the broad spectrum of their biological and pharmaceutical properties such as agricultural bactericide effects (Hideo, 1981), photodynamic therapy, anti-inflammatory activities (Poupelin *et al.*, 1978) and antiviral effects (Lambert *et al.*, 1997). In view of the importance of the title compound, (I), we report herein its crystal structure.

In the molecule of (I), (Fig. 1), rings A (C1—C6), B (C1/C6/O2/C13/C14/C17) and C (C9—C14) are not planar, having total puckering amplitudes, Q_T_, of 0.463 (3), 0.194 (3) and 0.459 (2) Å, respectively. They adopt envelope [φ = -2.43 (2)° and θ = 121.76 (3)°], boat [φ = 139.80 (3)° and θ = 88.77 (3)°] and envelope [φ = 176.89 (4)° and θ = 56.53 (3)°] conformations (Cremer & Pople, 1975). In rings A and C, atoms C4 and C12 displaced by -0.645 (3) and -0.641 (4) Å from the plane of the other ring atoms, respectively. Ring D (C18—C23) is, of course, planar.

In the crystal structure, intermolecular C—H···O hydrogen bonds (Table 1) link the molecules, generating centrosymmetric *R*_2_^2^(12), *R*_4_^4^(28) (Fig. 2) and *R*_2_^2^(16) (Fig. 3) ring motifs (Bernstein *et al.*, 1995; Etter, 1990), to form a three-dimensional network, in which they may be effective in the stabilization of the structure.

### Experimental

The syntheses of 2,2'-[(4-methoxyphenyl)methylene]bis(5,5-dimethylcyclohexane- 1,3-dione), (II), was achieved according to the procedure described in the literature (Horning & Horning, 1946). A mixture of (II) (5 mmol) and acetic acid (20 ml) was refluxed for 2 h. The reaction mixture was concentrated under reduced pressure and poured into crushed ice. The solid obtained was filtered off, dried and crystallized from EtOH-H_2_O (8:2) (yield: 92%, m.p. 515–517 K).

### Refinement

H atoms were positioned geometrically, with C—H = 0.93, 0.96, 0.97 and 0.98 Å for aromatic, methyl, methylene and methine H, respectively, and constrained to ride on their parent atoms with *U*_iso_(H) = 1.2*U*_eq_(C).

### Crystal data

**Table:**

C_24_H_28_O_4_	*Z* = 2
*M**_r_* = 380.46	*F*_000_ = 408
Triclinic, *P*1	*D*_x_ = 1.232 Mg m^−^^3^
Hall symbol: -P 1	Mo *K*α radiation λ = 0.71073 Å
*a* = 9.346 (5) Å	Cell parameters from 17059 reflections
*b* = 10.314 (5) Å	θ = 1.8–28.0º
*c* = 11.733 (5) Å	µ = 0.08 mm^−^^1^
α = 71.089 (5)º	*T* = 296 K
β = 84.253 (5)º	Prism, colorless
γ = 73.386 (5)º	0.66 × 0.54 × 0.41 mm
*V* = 1025.2 (9) Å^3^	

### Data collection

**Table:**

Stoe IPDSII diffractometer	4035 independent reflections
Monochromator: plane graphite	3450 reflections with *I* > 2σ(*I*)
Detector resolution: 6.67 pixels mm^-1^	*R*_int_ = 0.033
*T* = 296 K	θ_max_ = 26.0º
ω–scan rotation method	θ_min_ = 2.2º
Absorption correction: integration(X-RED32; Stoe & Cie, 2002)	*h* = −11→11
*T*_min_ = 0.947, *T*_max_ = 0.975	*k* = −12→12
17059 measured reflections	*l* = −14→14

### Refinement

**Table:**

Refinement on *F*^2^	Secondary atom site location: difference Fourier map
Least-squares matrix: full	Hydrogen site location: inferred from neighbouring sites
*R*[*F*^2^ > 2σ(*F*^2^)] = 0.041	H-atom parameters constrained
*wR*(*F*^2^) = 0.109	*w* = 1/[σ^2^(*F*_o_^2^) + (0.0512*P*)^2^ + 0.165*P*] where *P* = (*F*_o_^2^ + 2*F*_c_^2^)/3
*S* = 1.04	(Δ/σ)_max_ < 0.001
4035 reflections	Δρ_max_ = 0.16 e Å^−^^3^
253 parameters	Δρ_min_ = −0.17 e Å^−^^3^
Primary atom site location: structure-invariant direct methods	Extinction correction: none

### Special details

**Table:**

Geometry. All e.s.d.'s (except the e.s.d. in the dihedral angle between two l.s. planes) are estimated using the full covariance matrix. The cell e.s.d.'s are taken into account individually in the estimation of e.s.d.'s in distances, angles and torsion angles; correlations between e.s.d.'s in cell parameters are only used when they are defined by crystal symmetry. An approximate (isotropic) treatment of cell e.s.d.'s is used for estimating e.s.d.'s involving l.s. planes.
Refinement. Refinement of *F*^2^ against ALL reflections. The weighted *R*-factor *wR* and goodness of fit *S* are based on *F*^2^, conventional *R*-factors *R* are based on *F*, with *F* set to zero for negative *F*^2^. The threshold expression of *F*^2^ > σ(*F*^2^) is used only for calculating *R*-factors(gt) *etc*. and is not relevant to the choice of reflections for refinement. *R*-factors based on *F*^2^ are statistically about twice as large as those based on *F*, and *R*- factors based on ALL data will be even larger.

### Fractional atomic coordinates and isotropic or equivalent isotropic displacement parameters (Å^2^)

**Table:**

	*x*	*y*	*z*	*U*_iso_*/*U*_eq_	
O1	0.32183 (13)	0.81707 (14)	−0.08171 (10)	0.0796 (4)	
O2	0.55478 (9)	0.45682 (9)	0.26047 (8)	0.0459 (2)	
O3	0.03418 (11)	0.52647 (13)	0.26037 (11)	0.0718 (3)	
O4	0.02323 (12)	1.09853 (11)	0.34873 (10)	0.0672 (3)	
C1	0.42493 (13)	0.64720 (13)	0.09685 (11)	0.0431 (3)	
C2	0.43421 (15)	0.74638 (14)	−0.02413 (12)	0.0509 (3)	
C3	0.58637 (16)	0.75273 (16)	−0.07685 (12)	0.0551 (3)	
H3A	0.5799	0.8491	−0.1285	0.066*	
H3B	0.6149	0.6909	−0.1271	0.066*	
C4	0.70957 (15)	0.70997 (14)	0.01517 (12)	0.0471 (3)	
C5	0.70551 (13)	0.56537 (13)	0.10549 (12)	0.0452 (3)	
H5A	0.7443	0.4921	0.0669	0.054*	
H5B	0.7699	0.5444	0.1727	0.054*	
C6	0.55218 (13)	0.56175 (12)	0.15160 (11)	0.0407 (3)	
C7	0.68441 (19)	0.82180 (16)	0.07936 (15)	0.0632 (4)	
H7A	0.7614	0.7933	0.1378	0.076*	
H7B	0.5888	0.8309	0.1191	0.076*	
H7C	0.6872	0.9116	0.0215	0.076*	
C8	0.86223 (17)	0.69525 (18)	−0.04741 (15)	0.0649 (4)	
H8A	0.8782	0.6252	−0.0882	0.078*	
H8B	0.9388	0.6662	0.0115	0.078*	
H8C	0.8656	0.7851	−0.1048	0.078*	
C9	0.28957 (13)	0.50728 (13)	0.25682 (12)	0.0436 (3)	
C10	0.15563 (14)	0.46043 (16)	0.30671 (13)	0.0532 (3)	
C11	0.17414 (17)	0.32686 (18)	0.41176 (15)	0.0659 (4)	
H11A	0.1844	0.2477	0.3813	0.079*	
H11B	0.0839	0.3353	0.4606	0.079*	
C12	0.30751 (16)	0.29219 (16)	0.49231 (13)	0.0558 (3)	
C13	0.44636 (15)	0.29653 (14)	0.41151 (13)	0.0516 (3)	
H13A	0.5285	0.2921	0.4583	0.062*	
H13B	0.4743	0.2136	0.3838	0.062*	
C14	0.42175 (13)	0.42701 (13)	0.30557 (11)	0.0422 (3)	
C15	0.2821 (2)	0.4017 (2)	0.55902 (16)	0.0745 (5)	
H15A	0.2686	0.4950	0.5017	0.089*	
H15B	0.3672	0.3802	0.6082	0.089*	
H15C	0.1946	0.3988	0.6091	0.089*	
C16	0.3294 (2)	0.1439 (2)	0.58349 (18)	0.0846 (6)	
H16A	0.4154	0.1220	0.6317	0.102*	
H16B	0.3438	0.0751	0.5416	0.102*	
H16C	0.2426	0.1413	0.6345	0.102*	
C17	0.27361 (13)	0.64451 (13)	0.15483 (12)	0.0452 (3)	
H17	0.2064	0.6470	0.0947	0.054*	
C18	0.20586 (13)	0.77091 (13)	0.20118 (12)	0.0443 (3)	
C19	0.27809 (16)	0.79386 (16)	0.28732 (15)	0.0641 (4)	
H19	0.3717	0.7338	0.3138	0.077*	
C20	0.21497 (18)	0.90297 (17)	0.33441 (15)	0.0662 (4)	
H20	0.2659	0.9163	0.3920	0.079*	
C21	0.07566 (15)	0.99328 (13)	0.29641 (12)	0.0486 (3)	
C22	0.00317 (14)	0.97443 (13)	0.20907 (13)	0.0490 (3)	
H22	−0.0896	1.0355	0.1815	0.059*	
C23	0.06918 (13)	0.86400 (14)	0.16245 (12)	0.0473 (3)	
H23	0.0197	0.8523	0.1031	0.057*	
C24	−0.12136 (18)	1.19079 (18)	0.31659 (19)	0.0738 (5)	
H24A	−0.1932	1.1363	0.3366	0.089*	
H24B	−0.1240	1.2395	0.2315	0.089*	
H24C	−0.1448	1.2590	0.3598	0.089*	

### Atomic displacement parameters (Å^2^)

**Table:**

	*U*^11^	*U*^22^	*U*^33^	*U*^12^	*U*^13^	*U*^23^
O1	0.0601 (7)	0.0888 (8)	0.0657 (7)	−0.0011 (6)	−0.0250 (5)	−0.0010 (6)
O2	0.0324 (4)	0.0461 (5)	0.0500 (5)	−0.0061 (3)	−0.0027 (3)	−0.0058 (4)
O3	0.0375 (5)	0.0919 (8)	0.0881 (8)	−0.0172 (5)	−0.0057 (5)	−0.0291 (7)
O4	0.0681 (7)	0.0516 (6)	0.0810 (7)	0.0021 (5)	−0.0087 (5)	−0.0331 (5)
C1	0.0391 (6)	0.0435 (6)	0.0477 (7)	−0.0058 (5)	−0.0068 (5)	−0.0183 (5)
C2	0.0514 (7)	0.0497 (7)	0.0486 (7)	−0.0032 (6)	−0.0126 (6)	−0.0166 (6)
C3	0.0613 (8)	0.0545 (8)	0.0429 (7)	−0.0093 (6)	−0.0030 (6)	−0.0111 (6)
C4	0.0473 (7)	0.0454 (7)	0.0463 (7)	−0.0117 (5)	0.0010 (5)	−0.0123 (5)
C5	0.0368 (6)	0.0444 (7)	0.0490 (7)	−0.0054 (5)	−0.0006 (5)	−0.0120 (5)
C6	0.0385 (6)	0.0386 (6)	0.0441 (6)	−0.0068 (5)	−0.0025 (5)	−0.0141 (5)
C7	0.0657 (9)	0.0573 (9)	0.0751 (10)	−0.0215 (7)	0.0006 (7)	−0.0278 (7)
C8	0.0572 (9)	0.0680 (10)	0.0608 (9)	−0.0195 (7)	0.0102 (7)	−0.0090 (7)
C9	0.0363 (6)	0.0461 (7)	0.0539 (7)	−0.0085 (5)	−0.0008 (5)	−0.0248 (6)
C10	0.0391 (7)	0.0654 (8)	0.0660 (9)	−0.0161 (6)	0.0009 (6)	−0.0332 (7)
C11	0.0534 (9)	0.0766 (10)	0.0753 (10)	−0.0318 (8)	0.0059 (7)	−0.0232 (8)
C12	0.0480 (7)	0.0623 (8)	0.0571 (8)	−0.0193 (6)	0.0078 (6)	−0.0171 (7)
C13	0.0450 (7)	0.0470 (7)	0.0575 (8)	−0.0089 (6)	0.0049 (6)	−0.0138 (6)
C14	0.0357 (6)	0.0432 (6)	0.0507 (7)	−0.0095 (5)	0.0026 (5)	−0.0203 (5)
C15	0.0671 (10)	0.0969 (13)	0.0628 (10)	−0.0153 (9)	0.0069 (8)	−0.0372 (9)
C16	0.0777 (12)	0.0837 (12)	0.0802 (12)	−0.0335 (10)	0.0134 (9)	−0.0029 (10)
C17	0.0341 (6)	0.0495 (7)	0.0537 (7)	−0.0034 (5)	−0.0102 (5)	−0.0222 (6)
C18	0.0347 (6)	0.0447 (7)	0.0520 (7)	−0.0040 (5)	−0.0077 (5)	−0.0168 (5)
C19	0.0501 (8)	0.0606 (9)	0.0794 (10)	0.0128 (6)	−0.0310 (7)	−0.0339 (8)
C20	0.0646 (9)	0.0612 (9)	0.0744 (10)	0.0057 (7)	−0.0304 (8)	−0.0341 (8)
C21	0.0481 (7)	0.0390 (6)	0.0544 (7)	−0.0054 (5)	−0.0017 (6)	−0.0140 (5)
C22	0.0335 (6)	0.0427 (7)	0.0641 (8)	−0.0013 (5)	−0.0076 (5)	−0.0134 (6)
C23	0.0358 (6)	0.0490 (7)	0.0563 (7)	−0.0060 (5)	−0.0113 (5)	−0.0167 (6)
C24	0.0550 (9)	0.0597 (9)	0.1074 (14)	−0.0030 (7)	0.0101 (9)	−0.0406 (9)

### Geometric parameters (Å, °)

**Table:**

C1—C6	1.3380 (17)	C12—C16	1.527 (2)
C1—C2	1.4676 (19)	C12—C13	1.531 (2)
C1—C17	1.5103 (19)	C13—C14	1.4849 (19)
C2—O1	1.2149 (17)	C13—H13A	0.9700
C2—C3	1.503 (2)	C13—H13B	0.9700
C3—C4	1.528 (2)	C14—O2	1.3790 (16)
C3—H3A	0.9700	C15—H15A	0.9600
C3—H3B	0.9700	C15—H15B	0.9600
C4—C7	1.525 (2)	C15—H15C	0.9600
C4—C8	1.529 (2)	C16—H16A	0.9600
C4—C5	1.5325 (18)	C16—H16B	0.9600
C5—C6	1.4866 (18)	C16—H16C	0.9600
C5—H5A	0.9700	C17—C18	1.5214 (17)
C5—H5B	0.9700	C17—H17	0.9800
C6—O2	1.3779 (15)	C18—C23	1.3756 (17)
C7—H7A	0.9600	C18—C19	1.3843 (19)
C7—H7B	0.9600	C19—C20	1.370 (2)
C7—H7C	0.9600	C19—H19	0.9300
C8—H8A	0.9600	C20—C21	1.384 (2)
C8—H8B	0.9600	C20—H20	0.9300
C8—H8C	0.9600	C21—O4	1.3649 (16)
C9—C14	1.3373 (18)	C21—C22	1.3749 (19)
C9—C10	1.4693 (19)	C22—C23	1.3826 (19)
C9—C17	1.5083 (19)	C22—H22	0.9300
C10—O3	1.2205 (17)	C23—H23	0.9300
C10—C11	1.501 (2)	C24—O4	1.4170 (19)
C11—C12	1.529 (2)	C24—H24A	0.9600
C11—H11A	0.9700	C24—H24B	0.9600
C11—H11B	0.9700	C24—H24C	0.9600
C12—C15	1.525 (2)		
			
C6—C1—C2	118.25 (12)	C16—C12—C13	109.43 (13)
C6—C1—C17	122.42 (12)	C11—C12—C13	107.83 (13)
C2—C1—C17	119.33 (11)	C14—C13—C12	112.37 (11)
O1—C2—C1	120.64 (14)	C14—C13—H13A	109.1
O1—C2—C3	121.08 (13)	C12—C13—H13A	109.1
C1—C2—C3	118.22 (11)	C14—C13—H13B	109.1
C2—C3—C4	115.16 (12)	C12—C13—H13B	109.1
C2—C3—H3A	108.5	H13A—C13—H13B	107.9
C4—C3—H3A	108.5	C9—C14—O2	122.76 (12)
C2—C3—H3B	108.5	C9—C14—C13	125.86 (12)
C4—C3—H3B	108.5	O2—C14—C13	111.38 (10)
H3A—C3—H3B	107.5	C12—C15—H15A	109.5
C7—C4—C3	110.09 (12)	C12—C15—H15B	109.5
C7—C4—C8	109.28 (12)	H15A—C15—H15B	109.5
C3—C4—C8	110.25 (12)	C12—C15—H15C	109.5
C7—C4—C5	110.58 (12)	H15A—C15—H15C	109.5
C3—C4—C5	107.82 (11)	H15B—C15—H15C	109.5
C8—C4—C5	108.82 (11)	C12—C16—H16A	109.5
C6—C5—C4	112.35 (10)	C12—C16—H16B	109.5
C6—C5—H5A	109.1	H16A—C16—H16B	109.5
C4—C5—H5A	109.1	C12—C16—H16C	109.5
C6—C5—H5B	109.1	H16A—C16—H16C	109.5
C4—C5—H5B	109.1	H16B—C16—H16C	109.5
H5A—C5—H5B	107.9	C9—C17—C1	108.73 (10)
C1—C6—O2	122.58 (11)	C9—C17—C18	110.10 (11)
C1—C6—C5	125.87 (12)	C1—C17—C18	112.24 (10)
O2—C6—C5	111.55 (10)	C9—C17—H17	108.6
C4—C7—H7A	109.5	C1—C17—H17	108.6
C4—C7—H7B	109.5	C18—C17—H17	108.6
H7A—C7—H7B	109.5	C23—C18—C19	117.39 (12)
C4—C7—H7C	109.5	C23—C18—C17	121.70 (11)
H7A—C7—H7C	109.5	C19—C18—C17	120.88 (11)
H7B—C7—H7C	109.5	C20—C19—C18	121.55 (12)
C4—C8—H8A	109.5	C20—C19—H19	119.2
C4—C8—H8B	109.5	C18—C19—H19	119.2
H8A—C8—H8B	109.5	C19—C20—C21	120.15 (13)
C4—C8—H8C	109.5	C19—C20—H20	119.9
H8A—C8—H8C	109.5	C21—C20—H20	119.9
H8B—C8—H8C	109.5	O4—C21—C22	125.20 (12)
C14—C9—C10	118.31 (13)	O4—C21—C20	115.43 (12)
C14—C9—C17	122.32 (11)	C22—C21—C20	119.34 (12)
C10—C9—C17	119.36 (11)	C21—C22—C23	119.53 (12)
O3—C10—C9	120.22 (14)	C21—C22—H22	120.2
O3—C10—C11	121.56 (13)	C23—C22—H22	120.2
C9—C10—C11	118.17 (12)	C18—C23—C22	122.01 (12)
C10—C11—C12	115.00 (12)	C18—C23—H23	119.0
C10—C11—H11A	108.5	C22—C23—H23	119.0
C12—C11—H11A	108.5	O4—C24—H24A	109.5
C10—C11—H11B	108.5	O4—C24—H24B	109.5
C12—C11—H11B	108.5	H24A—C24—H24B	109.5
H11A—C11—H11B	107.5	O4—C24—H24C	109.5
C15—C12—C16	109.43 (15)	H24A—C24—H24C	109.5
C15—C12—C11	110.00 (13)	H24B—C24—H24C	109.5
C16—C12—C11	110.23 (14)	C6—O2—C14	117.94 (9)
C15—C12—C13	109.89 (13)	C21—O4—C24	117.83 (12)
			
C6—C1—C2—O1	173.50 (13)	C10—C9—C14—C13	−3.52 (19)
C17—C1—C2—O1	−6.3 (2)	C17—C9—C14—C13	175.26 (12)
C6—C1—C2—C3	−3.61 (18)	C12—C13—C14—C9	−23.36 (19)
C17—C1—C2—C3	176.58 (11)	C12—C13—C14—O2	157.27 (11)
O1—C2—C3—C4	156.50 (14)	C14—C9—C17—C1	17.55 (16)
C1—C2—C3—C4	−26.40 (18)	C10—C9—C17—C1	−163.68 (10)
C2—C3—C4—C7	−69.32 (16)	C14—C9—C17—C18	−105.79 (13)
C2—C3—C4—C8	170.04 (12)	C10—C9—C17—C18	72.98 (14)
C2—C3—C4—C5	51.38 (15)	C6—C1—C17—C9	−17.61 (16)
C7—C4—C5—C6	72.00 (14)	C2—C1—C17—C9	162.18 (11)
C3—C4—C5—C6	−48.39 (14)	C6—C1—C17—C18	104.44 (14)
C8—C4—C5—C6	−167.96 (12)	C2—C1—C17—C18	−75.76 (14)
C2—C1—C6—O2	−174.28 (11)	C9—C17—C18—C23	−116.51 (13)
C17—C1—C6—O2	5.52 (18)	C1—C17—C18—C23	122.23 (13)
C2—C1—C6—C5	5.41 (19)	C9—C17—C18—C19	61.45 (17)
C17—C1—C6—C5	−174.79 (11)	C1—C17—C18—C19	−59.82 (17)
C4—C5—C6—C1	22.49 (18)	C23—C18—C19—C20	1.4 (2)
C4—C5—C6—O2	−157.79 (10)	C17—C18—C19—C20	−176.62 (16)
C14—C9—C10—O3	−175.89 (13)	C18—C19—C20—C21	0.2 (3)
C17—C9—C10—O3	5.29 (19)	C19—C20—C21—O4	−179.83 (16)
C14—C9—C10—C11	1.51 (18)	C19—C20—C21—C22	−1.5 (2)
C17—C9—C10—C11	−177.32 (12)	O4—C21—C22—C23	179.39 (13)
O3—C10—C11—C12	−154.88 (14)	C20—C21—C22—C23	1.3 (2)
C9—C10—C11—C12	27.76 (19)	C19—C18—C23—C22	−1.7 (2)
C10—C11—C12—C15	68.08 (18)	C17—C18—C23—C22	176.34 (13)
C10—C11—C12—C16	−171.16 (14)	C21—C22—C23—C18	0.3 (2)
C10—C11—C12—C13	−51.75 (17)	C1—C6—O2—C14	9.04 (17)
C15—C12—C13—C14	−71.43 (16)	C5—C6—O2—C14	−170.69 (10)
C16—C12—C13—C14	168.39 (13)	C9—C14—O2—C6	−9.10 (17)
C11—C12—C13—C14	48.47 (16)	C13—C14—O2—C6	170.29 (10)
C10—C9—C14—O2	175.78 (11)	C22—C21—O4—C24	4.2 (2)
C17—C9—C14—O2	−5.43 (18)	C20—C21—O4—C24	−177.63 (15)

### Hydrogen-bond geometry (Å, °)

**Table:**

*D*—H···*A*	*D*—H	H···*A*	*D*···*A*	*D*—H···*A*
C11—H11A···O4^i^	0.97	2.57	3.364 (2)	139
C15—H15C···O3^ii^	0.96	2.58	3.506 (2)	161
C22—H22···O1^iii^	0.93	2.42	3.343 (2)	171

Symmetry codes: (i) *x*, *y*−1, *z*; (ii) −*x*, −*y*+1, −*z*+1; (iii) −*x*, −*y*+2, −*z*.
